# Development of a Structured Query Language and Natural Language Processing Algorithm to Identify Lung Nodules in a Cancer Centre

**DOI:** 10.3389/fmed.2021.748168

**Published:** 2021-11-04

**Authors:** Benjamin Hunter, Sara Reis, Des Campbell, Sheila Matharu, Prashanthi Ratnakumar, Luca Mercuri, Sumeet Hindocha, Hardeep Kalsi, Erik Mayer, Ben Glampson, Emily J. Robinson, Bisan Al-Lazikani, Lisa Scerri, Susannah Bloch, Richard Lee

**Affiliations:** ^1^The Royal Marsden National Health Service (NHS) Foundation Trust, Lung Unit, London, United Kingdom; ^2^Department of Surgery and Cancer, Imperial College London, London, United Kingdom; ^3^Imperial College Healthcare Trust, Respiratory Medicine, London, United Kingdom; ^4^Imperial College Healthcare National Health Service (NHS) Trust, Imperial Clinical Analytics, Research and Evaluation, London, United Kingdom; ^5^The Royal Marsden National Health Service (NHS) Foundation Trust, Royal Marsden Clinical Trials Unit, London, United Kingdom; ^6^The Institute for Cancer Research, Computational Biology and Chromogenetics, London, United Kingdom; ^7^Imperial College London, National Heart and Lung Institute, London, United Kingdom; ^8^The Institute for Cancer Research, Early Diagnosis and Detection, Genetics and Epidemiology, London, United Kingdom

**Keywords:** lung nodule, informatics, structured query language (SQL), natural language processing (NLP), machine learning

## Abstract

**Importance:** The stratification of indeterminate lung nodules is a growing problem, but the burden of lung nodules on healthcare services is not well-described. Manual service evaluation and research cohort curation can be time-consuming and potentially improved by automation.

**Objective:** To automate lung nodule identification in a tertiary cancer centre.

**Methods:** This retrospective cohort study used Electronic Healthcare Records to identify CT reports generated between 31st October 2011 and 24th July 2020. A structured query language/natural language processing tool was developed to classify reports according to lung nodule status. Performance was externally validated. Sentences were used to train machine-learning classifiers to predict concerning nodule features in 2,000 patients.

**Results:** 14,586 patients with lung nodules were identified. The cancer types most commonly associated with lung nodules were lung (39%), neuro-endocrine (38%), skin (35%), colorectal (33%) and sarcoma (33%). Lung nodule patients had a greater proportion of metastatic diagnoses (45 vs. 23%, *p* < 0.001), a higher mean post-baseline scan number (6.56 vs. 1.93, *p* < 0.001), and a shorter mean scan interval (4.1 vs. 5.9 months, *p* < 0.001) than those without nodules. Inter-observer agreement for sentence classification was 0.94 internally and 0.98 externally. Sensitivity and specificity for nodule identification were 93 and 99% internally, and 100 and 100% at external validation, respectively. A linear-support vector machine model predicted concerning sentence features with 94% accuracy.

**Conclusion:** We have developed and validated an accurate tool for automated lung nodule identification that is valuable for service evaluation and research data acquisition.

## Introduction

The evaluation and stratification of incidental lung nodules places a large resource burden on health services. Current British Thoracic Society guidelines require serial computed-tomography (CT) scans to demonstrate growth (1), which places a demand on limited radiology services and generates anxiety for patients. Numerous research studies are exploring methods for improving risk-stratification through computational methods, including radiomics and deep-learning (2, 3), but for trusts to improve existing pathways, the first step is to evaluate local services to gauge the scale of the problem. Methods for tracking and following-up lung nodules vary widely, and methods to identify such patients rapidly and accurately are needed. In addition, there is limited data on how such nodules are managed in the context of pre-existing cancer, with studies suggesting a range of possible clinical entities aside from pulmonary metastases (4).

Many studies have demonstrated the utility of text-based algorithms for medical data curation, both with traditional machine-learning and deep-learning architectures (5–8). A 2012 study by Danforth et al. demonstrated that natural language processing (NLP) approaches are effective at identifying patients with lung nodules in the community, reporting a sensitivity and specificity of 96 and 86%, respectively (9). The use of ICD-9 codes to identify transcripts is a possible drawback, which may have led to incomplete data with an over-representation of larger nodules (9). Nevertheless, the algorithm showed consistent performance across healthcare systems at external validation (10). A similar approach was adopted by Kang et al., who report 91 and 82% sensitivity and specificity for nodule detection (11).

In recent years, a growing number of studies have utilised medical images directly for data extraction and curation, some of which arose from ImageCLEFmed challenges (12). For example, Fushman et al. found that combining text and imaging data improved the accuracy of data retrieval when compared to either modality alone (13). Zhou et al. developed a deep-learning architecture, “3DSE,” to identify and extract dynamic CT liver scans and classify them according to contrast phase, which outperformed text-mining approaches (14). In the lung nodule setting, a hybrid model incorporating deep-learning computer-vision and CT-report NLP was able to identify nodules missed by text-only identification, albeit at the expense of additional false-positives (15). It is worth noting that while multi-modal approaches may improve accuracy, they may also be more computationally intensive, data hungry or logistically challenging to implement (16).

It is recognised that many algorithms in the literature still require considerable human-input due to low specificities, and other criticisms, including a lack of statistical powering and external validation, can be made (17).

In this study, we developed a structured-query-language (SQL) algorithm to identify patients with pulmonary nodules at a tertiary cancer centre, which was validated at an external university hospital. Natural language processing (NLP) and machine learning methodology were then used to categorise lung nodule reports based on sentence features. The intended use of this algorithm is to facilitate research and service evaluation of lung nodule clinical pathways.

### Hypotheses

1) An algorithm can be developed to identify lung nodule CT scans with high clinician agreement and accuracy (defined as Krippendorf's alpha > 0.85 and sensitivity/specificity > 0.9).
2) A machine-learning classifier can be trained to predict concerning nodule features with a minimum AUC > 0.65.

## Methods

### Study Design

This retrospective observational study/multi-centre service evaluation was performed with approval from the Royal Marsden Hospital (SE1018) and Imperial College London Healthcare service evaluation boards. The range of CT dates included was from 26th October 2011 to 24th July 2020. All patients with CT scan reports between these dates were eligible for inclusion. A 10-year study period was used to give a representative estimate of the nodule population at the hospital. The algorithm was developed iteratively over two cycles, and the final version was applied on 24th July 2020.

### Data Processing and Algorithm Development

**Figure 1:**
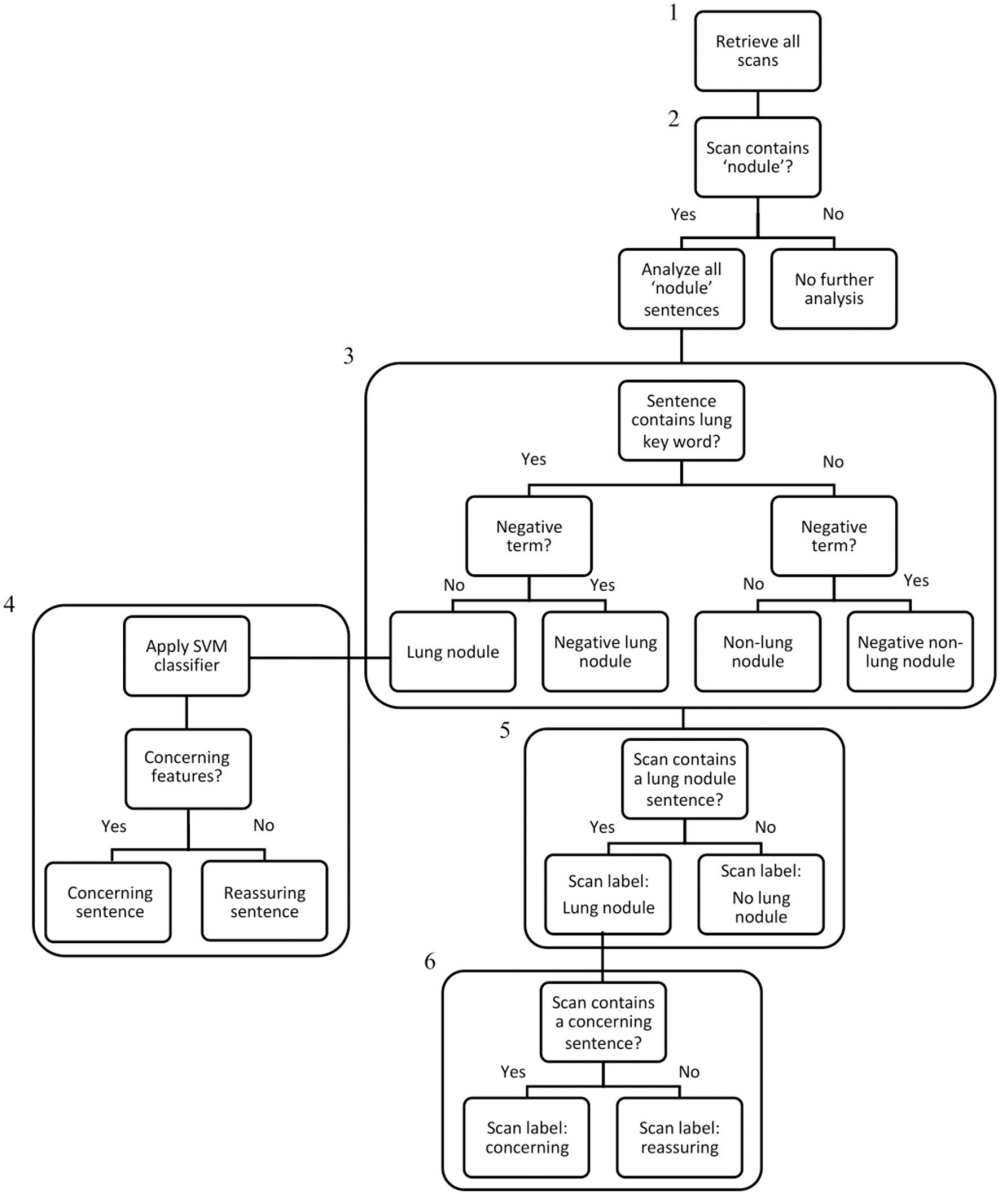
Algorithm data processing flowchart. Step 1: All CT scan reports generated in the 10-year study period were extracted from the EHR system using an SQL search (242,996 scans – the “denominator” set). Step 2: The NLP algorithm searched all scan reports for the term “nodule,” and if present, selected the scans for analysis (79,534 scans). Step 3: All sentences within nodule scans were analysed and categorised individually and assigned a label: “Lung nodule,” “Negative Lung Nodule,” “Non-Lung Nodule,” “Negative Non-Lung Nodule”. Step 4: For sentences categorised as “Lung Nodule,” the linear-SVM classifier was used to assign a binary classification of “concerning” or “reassuring.” Step 5: If any sentence within a scan contained a “Lung Nodule” categorisation, the scan received a “Lung Nodule” label. Step 6: If any “Lung Nodule” scan contained a concerning sentence, the scan received a “concerning” label. As scans without any nodule terms were excluded at Step 2, the IDs of lung nodule patients were used to ensure all their scans from the denominator set (including scans without any nodules terms) were included in the final lung nodule dataset of 14,586 patients and 110,339 scans.

The Royal Marsden Hospital maintains an integrated data warehouse of electronic patient record (EPR) data. We created a rule-based SQL algorithm to extract data from multiple tables within the EPR repository, which were aligned to Online Transaction Processing (OLTP) constructs. The algorithm data processing pipeline is displayed in Figure 1.

Firstly, all CT scan reports generated within the study period (242,996 scans, the “denominator set”) were obtained using the SQL-search. Patient cancer diagnoses were extracted from clinical-coder entries and processed into disease groups by searching for a pre-specified list of terms within the text using the “grepl” function in R (Supplementary Table 1). Patients with ambiguous or “unknown” anatomical origin sites were grouped as “Other”; those with missing disease group data were deemed “Missing” or “Metastatic Not Otherwise Specified (NOS)” according to metastatic status. A second primary cancer status was attributed if an additional diagnostic code was identified (excluding “missing” or “metastatic NOS” codes).

All NLP code was developed using Python v3.8. The second algorithm step was to identify scans containing the word “nodule” using a search of the report text after converting to lower case and removing whitespaces and line breaks (79,534 scans).

Report findings and opinion sections were identified and divided into component sentences using regex tokenisation. We identified CT reports containing pulmonary nodules using the terms “pulmonary,” “lung,” “lingula,” “upper lobe,” “lower lobe,” “middle lobe,” and “subpleural” to indicate lung origin. pyConTextNLP was used to search parsed sentences for modifying terms that may alter the meaning, such as “no lung nodules,” within an external yml list. Sentences were categorised as: Lung Nodule, Negative Lung Nodule, Non-lung nodule, Negative Non-lung nodule. Scans were assigned a “Lung Nodule” status if any component sentence was labelled “Lung Nodule,” and patients with any “Lung Nodule” scans received a patient-level status of “Lung Nodule.”

As non-nodule scans were initially excluded by algorithm step 2 (Figure 1), the IDs of patients with lung nodules were cross-referenced with the denominator set, to include all scans regardless of nodule status for those with a patient-level lung nodule attribution. The final lung nodule dataset consisted of 14,586 patients and 110,339 scans.

### Data Access

At the primary site, extracted data were link-anonymised and uploaded to the Biomedical Research Informatics Digital Environment (Aridhia Informatics Limited) for access by co-investigators via user-specific accounts. The original, de-identified data is housed in a password-protected master-spreadsheet with user-specific access on a hospital shared network-drive. Data were not shared between the primary and external-validation centres.

### Machine Learning Models

Two clinicians (BH and HK) assigned a binary label of “concerning” or “reassuring” to 2,000 lung nodule sentences. Reassuring sentences were those containing stable nodules, shrinking nodules, nodules described by the reporting radiologist as likely benign or nodules with no concerning features. The concerning sentences consisted of nodules that were growing, had concerning morphology, or that the reporting radiologist suggested were suspicious or likely metastatic (except where such features were previously reported and unchanged).

Inter-observer agreement between the clinicians was 0.79, and discrepancies were resolved in 177 cases by deferral to senior opinion (RL). Using these clinical labels as ground-truth, machine-learning algorithms were trained to predict concerning status.

A corpus of words was generated using the R package tm. Sentences were transformed to lower case, numbers and punctuation were removed, and words were converted to their stems using SnowBallC. Sparse terms (<0.1%) and stopwords were removed. A sparse-matrix was then generated as input variables for the machine-learning models. Models were trained using randomForest, stats, e1071, naivebayes, and XGBoost packages. Default hyper-parameters were used except for the following specified settings:

- Random Forest: number of trees: 128.
- XGBoost: max depth: 2, learning rate: 1, number of threads: 2, number of rounds: 2, objective: binary logistic regression.
- Support-Vector Machine: type: C-classification, kernel: linear.

Data were randomly split into training (*n* = 1,332) and test sets (*n* = 668) using a 2:1 ratio. The proportion of concerning cases was 25% in both the training and test sets. The best performing model (linear-SVM) was used to assign a suspicious flag to all lung nodule CT scan reports with concerning features in the dataset.

### Statistical Analysis and Power Calculations

Statistical oversight was provided by statisticians and bioinformaticians at the Royal Marsden Hospital and Institute for Cancer Research, respectively. Analysis was performed using R Studio v1.4.1717. Significance levels were set at 0.05 and *P*-values are presented unadjusted. Formal comparisons of proportions and means were performed using Chi-Squared and student's *t*-tests, respectively.

The R package kappaSize was used to generate sample size estimates for inter-reader reliability. Assuming a kappa statistic of 0.85, and defining acceptable precision as a confidence interval of width 0.2 (0.75–0.95), the minimum sample size required was 116. Scoring all sentences for a random selection of 116 patients lead to evaluation of 182 sentences. Krippendorf's-alpha values for inter-rater reliability matrices were calculated using the irr package.

Caret was used for calculation of the sensitivity, specificity, positive-predictive value (PPV), and negative-predictive value (NPV). For classification models, the R package pROC was used to calculate sample size estimates and generated AUC/ROC curves. Ninety percentage power to detect an AUC of 0.65 would require 52 cases and 104 controls.

### Validation

The first iteration classified sentences as pertaining to lung nodules (Yes or No) and as positive or negative statements, using “lung” to denote organ of origin. Manual validation was performed by a post-fellowship Clinical Oncology Registrar (BH, Rater 1) and a Consultant Respiratory Physician (RL, Rater 2) in a random sample of 150 patients. Both reviewers were blinded to the algorithm results.

Amendments were made to include additional nodule terms and to group categorisations into one of the four composite groups. Repeat manual validation was performed by BH (Rater 1) and a Clinical Oncology Registrar (SH, Rater 2) in 182 sentences.

A random selection of 500 CT scan reports from the included period were manually reviewed to check all scans and sentences within the scans pertaining to lung nodules were identified.

The algorithm was externally validated at Imperial College London Healthcare Trust, which includes general, non-cancer health services. Data were curated and anonymised in the Imperial Clinical Analytics, Research and Evaluation system to match the Royal Marsden Hospital specification. Specifically, python scripts were used to change data frames based on the external data schema and to feedback results into HIVE tables, and the regex tokenizer was altered to identify centre-specific scan report text start and stop delimiters. Manual external validation was performed in 142 patients by a Respiratory Medicine Registrar (PR).

## Results

### Patient Characteristics

We identified a denominator set of 59,931 patients with 242,996 CT scans performed between 31st October 2011 and 24th July 2020. The SQL algorithm extracted a cohort of 27,895 patients and 79,534 scans containing nodule terms (any anatomical subsite), which were broken-down into 126,685 sentences for classification. 51,214 sentences (40%) referred to lung nodules, 32,056 (25%) referred to negative lung nodules (scan reports explicitly stating absence of lung nodules), 36,214 (29%) referred to non-lung nodules (nodules in non-lung anatomical sites), and 7,201 (6%) referred to negative non-lung nodules. Sentence attributions were combined into a scan and patient-level nodule status readout. This final lung nodule dataset consisted of 14,586 patients and 110,339 scans.

**Figure 2:**
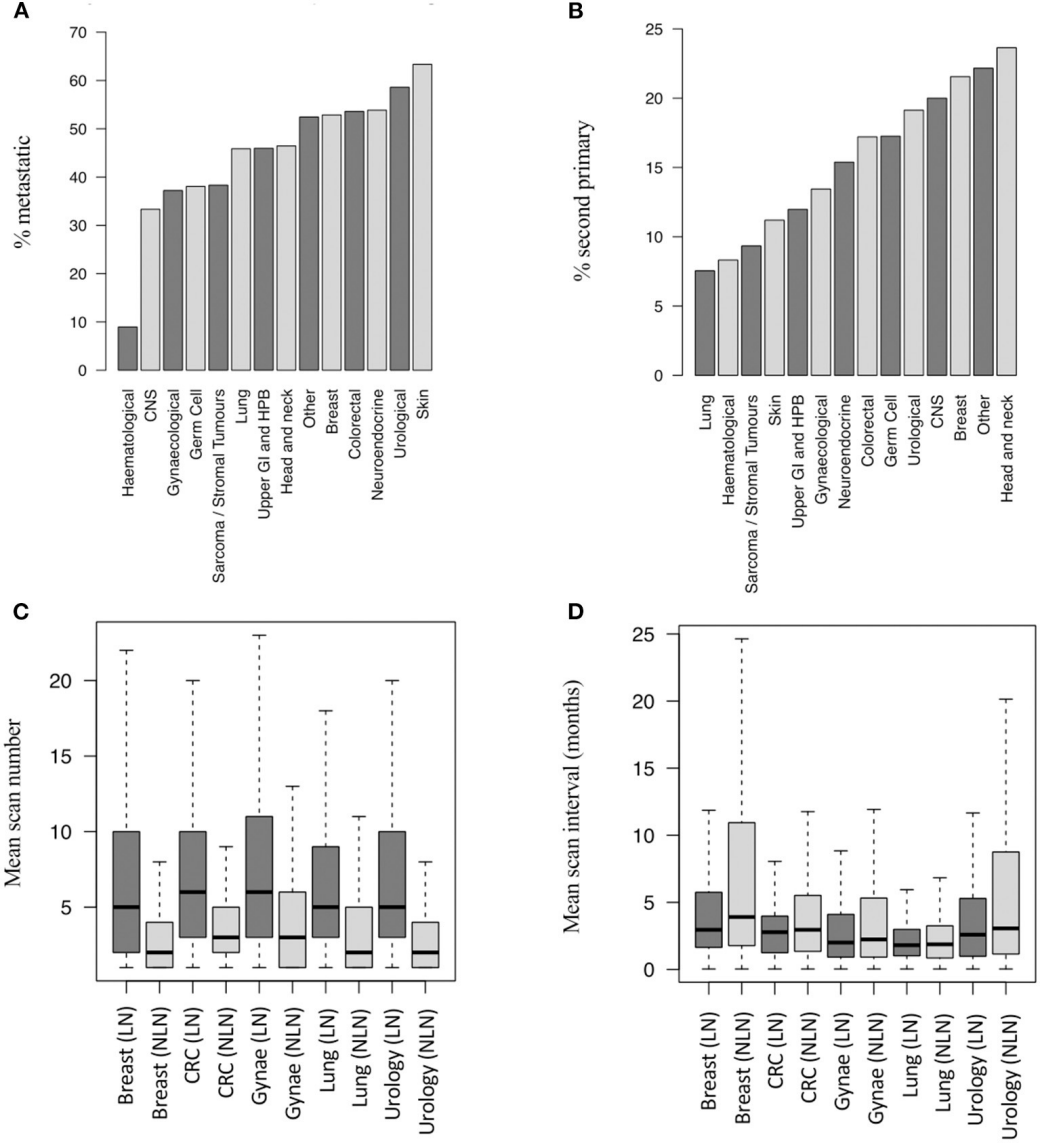
Metastatic and second primary status in lung nodule patients. **(A)** Proportion of LN patients with metastatic disease by cancer group (14,586 patients). **(B)** Proportion of LN patients with SPs by cancer group (14,586 pts). **(C)** Number of post-baseline scans per patient by LN status and disease group (19,779 pts – top 5 disease groups by scan number shown). **(D)** Scan interval by LN status and disease group (19,779 pts - top 5 disease groups by scan number shown). SP, second primary; LN, lung nodule; NLN, no lung nodule; CNS, central nervous system; CRC, colorectal cancer; Gynae, gynaecological cancer; Haem, haematological cancer; N.E, neuro-endocrine cancer; UGI/HPB, upper gastro-intestinal/hepatobiliary cancer.

The generated database allowed assessment of the surveillance, second primary and metastatic status for lung nodule patients (Table 1; Figure 2). The primary cancer types most commonly associated with lung nodules were lung (39%), neuro-endocrine (38%), skin (35%), colorectal (33%) and sarcoma (33%). As anticipated, a higher proportion of patients with lung nodules had a coded diagnosis of metastatic disease than those without [6,528 out of 14,586 patients (45%) vs. 10,413 out of 45,345 patients (23%), respectively, *p* < 0.001] (Figure 2).

**Table 1:** Summary of lung nodule and metastatic status amongst primary cancer groups.

**Disease group**	***n***	***n* Lung nodules(% of disease group total)**	***n* Lung nodules and metastases (% of lung nodules)**
Breast	7,364	1,617 (22)	851 (53)
CNS	551	17 (3)	5 (29)
Colorectal	5,556	1,819 (33)	872 (48)
Germ Cell	1,387	256 (18)	86 (34)
Gynaecological	6,299	1,683 (27)	537 (32)
Haematological	3,782	862 (23)	71 (8)
Head and neck	1,915	492 (26)	222 (45)
Lung	4,445	1,751 (39)	729 (42)
Metastatic NOS	664	219 (33)	219 (100)
Missing	8,352	441 (5)	218 (49)
Neuroendocrine	39	15 (38)	8 (53)
Other	1,839	421 (23)	211 (50)
Sarcoma	3,336	1,117 (33)	403 (36)
Skin	2,976	1,035 (35)	649 (63)
Upper GI/HPB	4,446	1,246 (28)	534 (43)
Urology	6,980	1,595 (23)	921 (58)

*CNS, central nervous system; NOS, Not otherwise specified; GI/HPB, Gastro-intestinal/hepatobiliary*.

The tumour types most commonly associated with co-diagnoses of lung nodules and metastatic disease were skin (63%), urology (58%), breast (53%), neuro-endocrine (53%), and other (50%) (Figure 2A). Similarly, lung nodules were more commonly associated with second primary cancers than non-lung nodule cases [2,049 out of 14,586 patients (14%) vs. 3,691 out of 45,345 patients (8%), respectively, *p* < 0.001]. The tumour subtypes most commonly associated with lung nodules and second primary cancers (any subsite) were head and neck (24%), other (22%), breast (21%), CNS (20%), and urological (19%) cancers (Figure 2B).

The mean number of post-baseline CT scans was higher in patients with lung nodules than those without (6.56 vs. 1.93, *p* < 0.001), and the scan interval time was shorter (4.1 vs. 5.9 months, *p* < 0.001). Figures 2C,D show the difference in scan number and frequency for the five cancer types contributing the highest number of CT scans to the dataset.

**Figure 3:**
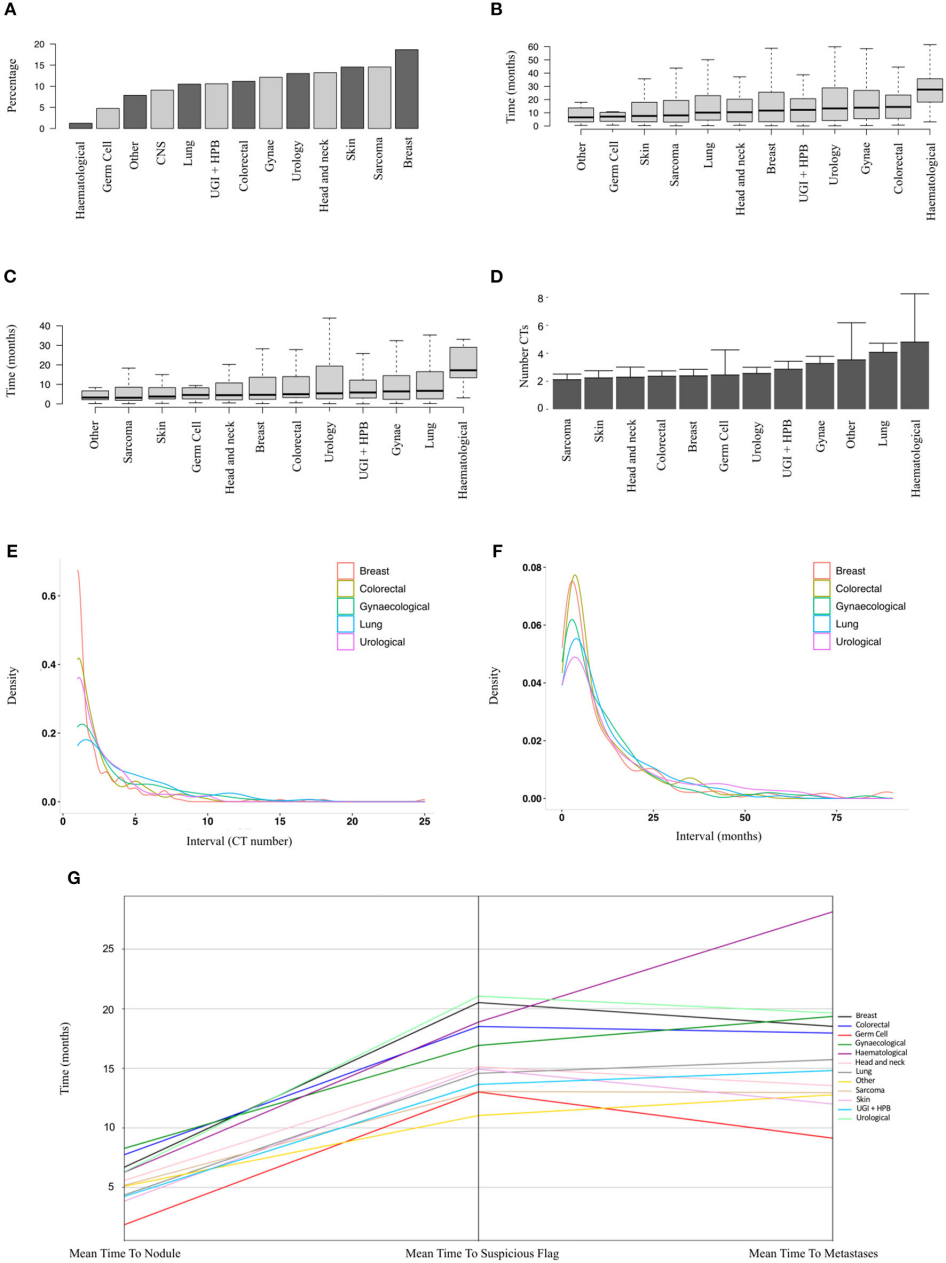
Sub-group analysis of patients developing metastatic disease after a nodule diagnosis. **(A)** Proportions of patients developing metastatic disease by tumour group (9,077 patients, ranked by median). **(B)** Time to earliest metastasis in months from study inclusion by disease group (1,019 patients, ranked by median). **(C)** Time to earliest metastasis in months from lung nodule by disease group (1,019 patients, ranked by median). **(D)** Mean number of CT scans (with upper 95% CIs) between nodule diagnosis and metastases by tumour group (1,019 patients, ranked by mean). **(E,F)** Frequency density plots showing the number of CTs and time in months, respectively, between nodule diagnosis and metastasis by disease group (5 groups of 1,019 patients shown). **(G)** Parallel coordinates plot showing the mean time in months to lung nodule, suspicious flag (as assigned by the linear-SVM machine-learning model) or metastasis by disease group (1,019 patients).

In order to identify patients where the lung nodule may have represented or co-arisen with metastatic disease, we performed analysis on a subset of patients who did not have a metastatic diagnosis prior to their nodule diagnosis (*n* = 9,077 patients, of whom 1,019 developed metastatic disease). Figure 3A shows the relative proportions of patients developing metastatic disease after a nodule diagnosis by disease group. The proportion was highest for breast cancer patients (19%) and lowest for haematology patients (1%).

The mean time from first available scan to a metastatic diagnosis, and from lung nodule detection to a metastatic diagnosis, is shown in Figures 3A,B for the various disease groups. As well as a lower proportion of patients with nodules developing metastases, haematological patients also had the longest duration from baseline or first lung nodule scan to metastasis (median 27.5 and 17.2 months, respectively). Of particular note, the median interval from a nodule diagnosis to metastatic disease was around 6 months or less for all solid tumour groups (Figure 3C). For sarcoma, skin, head and neck, colorectal breast and germ cell carcinoma, there was a mean of only two scans between a nodule diagnosis and metastatic disease (Figure 3D). The frequency density plots for the interval number of CTs or interval time in months from nodule to metastatic disease are shown in Figures 3E,F, respectively, and show a positively skewed distribution, but consistent clustering of frequencies around the early intervals with similar timings for all cancer types. We used parallel co-ordinates plots (Figure 3G) to map the sequence of events from nodule to our “suspicious” classifier to metastases in this cohort. In five tumour types, the transition to a suspicious classification preceded the development of metastases. The proportion of patients with a suspicious classifier before or after developing metastases by disease group is shown in Supplementary Table 1 and Supplementary Figure 1.

### Nodule Search Tool Performance

Evaluation of a random selection of 500 CT scan reports from within the 10-year denominator set showed that our model identified nodule reports, and the number of sentences within each report, containing lung nodule terms with 100% accuracy.

Manual validation of lung nodule sentence classification was performed for the first and second iteration of the algorithm by two clinicians in 150 and 182 sentences, respectively. The results are summarised in Table 2. There was high inter-reader reliability (Krippendorf's alpha > 0.90) between clinicians and the algorithm and individual observers in both iterations.

**Table 2:** Sentence classification inter-reader agreement.

**Validation 1**	
Lung nodule (Yes or No)	Krippendorf's Alpha
Algorithm and Rater 1	0.94
Algorithm and Rater 2	0.91
Rater 1 and Rater 2	0.98
Positive or negative sentence	Krippendorf's Alpha
Algorithm and Rater 1	0.90
Algorithm and Rater 2	0.94
Rater 1 and Rater 2	0.98
**Validation 2**	
Sentence group	Krippendorf's Alpha
Algorithm and Rater 1	0.92
Algorithm and Rater 2	0.93
Rater 1 and Rater 2	0.95
**External validation**	
Sentence group	Krippendorf's Alpha
Algorithm and Rater 1	0.98

The algorithm was highly accurate at identifying lung nodule sentences (Table 3). Accuracy for nodule sentence detection was 0.96 (0.9224–0.9844) at internal validation, and 1.0 (0.9744–1) at external validation.

**Table 3:** Performance metrics for identification of lung nodules.

**Metric**	**Internal validation**	**External validation**
Accuracy	0.96 (95% CI: 0.9224, 0.9844)	1.00 (95% CI 0.9744, 1)
**Lung nodule identification**		
Sensitivity	0.99	1.00
Specificity	0.94	1.00
PPV	0.93	1.00
NPV	0.94	1.00

*PPV, positive predictive value; NPV, negative predictive value*.

### Machine-Learning Models

Our sparse matrix, consisting of 480 words taken from 2,000 sentences, was used to train five classification algorithms to predict clinically concerning changes in lung nodule scan reports. A summary of model performance in the test set (668 cases) is provided in Table 4. The Linear Support Vector Machine (SVM) performed best, with an accuracy, sensitivity and specificity of 0.94, 0.90, and 0.96, respectively.

**Table 4:** Performance of machine-learning classifiers for concerning status.

**Method**	**Accuracy (95% CI)**	***p***	**F1**	**Sensitivity**	**Specificity**	**PPV**	**NPV**
Logistic regression	0.82 (0.79–0.85)	<1 × 10^−5^	0.71	0.79	0.83	0.64	0.91
XG boost (LR)	0.88 (0.85–0.90)	<1 × 10^−5^	0.76	0.70	0.95	0.84	0.95
Naïve Bayes	0.90 (0.88–0.92)	<1 × 10^−5^	0.83	0.86	0.92	0.80	0.94
Random forest	0.94 (0.91–0.95)	<1 × 10^−5^	0.88	0.88	0.96	0.89	0.95
Linear SVM	0.94 (0.92–0.95)	<1 × 10^5^	0.89	0.90	0.96	0.88	0.96

Recursive feature elimination (RFE) revealed that the top-ten weighted features in the SVM-model were the following word stems: still, resolut, promin, larger, increas, less, conspicu, subsequ, eight, decreas.

## Discussion

Numerous studies have suggested that indeterminate lung nodules are an increasingly common finding (18), but few have looked at the scale of the problem in cancer centres, where CT scans are widely used for diagnosis, response assessment and post-treatment surveillance. We have described for the first time the breadth and complexity of this cohort in a tertiary cancer centre, highlighting that nodules are both extremely common, and may represent a broad span of clinical entities aside from metastatic disease. Indeed, although nodules patients were proportionally twice as likely to develop metastatic disease, over half did not within a mean surveillance period of 18 months (range 0–104 months), suggesting that careful evaluation is needed before assuming a metastatic aetiology. The observation that patients with lung nodules receive a greater number of scans, and at shorter intervals, may reflect their inherent complexity, and could be important when planning dedicated nodule services.

It appears that lung nodule aetiology differs significantly by primary cancer group, which may lead to further research into personalised surveillance according to cancer history. A higher proportion of patients with breast cancer went on to develop metastatic disease following a lung nodule diagnosis. The rate was lowest for haematological cancers, where alternative aetiological factors such as infection or post-treatment changes may be more likely. In addition, we report the novel finding that the interval between a nodule diagnosis and development of metastatic disease is frequently within two scans for many tumour groups, which may present a unique window of diagnostic opportunity.

Our SQL and NLP algorithm is highly accurate, and we have demonstrated that it can be adapted for deployment at external centres while maintaining high performance. As health-services move towards national lung cancer screening programmes, and as many centres seek to rationalise their nodule surveillance services, such a tool could be highly useful for local service evaluation and research dataset creation.

It is likely that a key component of future healthcare automation will revolve around computer-physician cross-talk, particularly alerting doctors to changes which may require urgent clinical action. Our algorithm could underpin a flag system, which interprets clinical report text and highlights concerning features, and is a novel application of NLP technology with excellent accuracy. Although this requires further development and validation before it could be used clinically, we have demonstrated the utility of this approach.

There are some important limitations of this work to consider. Firstly, disease group attribution was based on coded-diagnoses in patient EPRs, but some variability of entry terms was identified, including alternate spellings of disease sites (oesophageal vs. esophageal), abbreviations (OGJ, O-G, OJG), misspellings/typos, ambiguous or missing anatomical data (ie adenocarcinoma NOS, squamous cell cancer chest) or missing entries. Such variability could have introduced inaccuracy of disease group attribution, though groupings were inspected manually to guard against this, and the vast majority had non-missing headings.

It is also important to consider that in many cases, our clinical findings relate to correlation rather than causality. For example, although we have shown proportional differences in the likelihood of a nodule-metastasis co-diagnosis, the current model does not have the degree of granularity required to establish metastatic aetiology. This would require further model refinement and manual case-note review. One should also note that our data are reported for all stage-groups together, but differential rates of lung-nodule and metastatic co-diagnoses by stage may exist between disease groups, which is not provided in this present study. We also acknowledge that nodule count, size and location are not captured by our current model, and may be avenues for further development.

One further criticism could be that our models were developed in a tertiary cancer centre rather than a generalist hospital, which could limit applicability. In answer, we have demonstrated that performance was sustained at an external university hospital which is not a dedicated cancer hospital, but recognise that further external testing would be required before clinical use could be considered.

The presented model identifies nodules based on a list of pre-specified terms, agreed by consensus discussion with respiratory physicians, to best describe nodules anatomically attributable to the lung. There are certain contexts where a human can infer lung attribution, i.e., “the nodule is amenable to cardio-thoracic surgery,” which would not be identified by this hand-crafted approach, and our choice of anatomic terms includes an element of subjectivity. Nevertheless, the performance at internal and external validation was highly promising.

In conclusion, we have developed and externally validated a novel algorithm to identify lung nodules with high accuracy, and described for the first time the lung-nodule landscape in a tertiary cancer centre.

## Data Availability Statement

The datasets presented in this article are not readily available because we do not have permissions to allow sharing of the patient data that contributed to this study.

## Ethics Statement

These studies involving human participants were reviewed and approved by the Royal Marsden Hospital (SE1018) and Imperial College London Healthcare service evaluation boards. Written informed consent for participation was not required for this study in accordance with the national legislation and the institutional requirements.

## Author Contributions

BH contributed to algorithm design, performed the data analysis, generated the figures and wrote the manuscript. SR and DC developed and ran the NLP algorithm. SM performed anonymisation, incorporated data into the online environment and performed checks on analyses. PR, SH, and HK assisted with validation experiments. LM and BG adapted and ran the algorithm at the external centre. EM and SB supervised external validation. ER and BA-L provided statistical and informatics support. LS provided managerial and project support for the Biomedical Research Informatics Digital Environment. RL generated the initial project idea, contributed to algorithm design, provided intellectual input and supervision of this work and contributed to manuscript preparation. All authors contributed to the article and approved the submitted version.

## Funding

This project represents independent research funded by: (1) Royal Marsden Partners Cancer Alliance, (2) the Royal Marsden Cancer Charity, (3) the National Institute for Health Research (NIHR) Biomedical Research Centre at the Royal Marsden NHS Foundation Trust and The Institute of Cancer Research, London, (4) Cancer Research U.K. (C309/A31316) and (5) United Kingdom Research and Innovation Centre for Doctoral Training in Artificial Intelligence for Healthcare (P/S023283/1). (6) This research was supported by the Imperial Clinical Analytics Research and Evaluation (iCARE) environment and used the iCARE team and data resources (https://imperialbrc.nihr.ac.uk/facilities/icare/). The research was supported by the National Institute for Health Research (NIHR) Imperial Biomedical Research Centre (BRC).

## Author Disclaimer

The views expressed are those of the authors and not necessarily those of the NHS, the NIHR or the Department of Health and Social Care.

## Conflict of Interest

BA-L is an employee of the Institute of Cancer Research (ICR) which operates a Rewards to Inventors scheme through which employees of the ICR may receive financial benefit following commercial licencing. She has received honoraria or consultancy fees from GSK and Definiens AG (now part of the Astra Zenica group). RL is funded by the Royal Marsden NIHR BRC and Royal Marsden Cancer charity. RL's institution receives compensation for time spent in a secondment role for the lung health check program. He has received research funding from CRUK, Innovate UK (co-funded by Roche and Optellum), RM Partners Cancer Alliance and NHSX (co-applicant in grants with Optellum and Aidence). He has received honoraria from CRUK. The remaining authors declare that the research was conducted in the absence of any commercial or financial relationships that could be construed as a potential conflict of interest.

## Publisher's Note

All claims expressed in this article are solely those of the authors and do not necessarily represent those of their affiliated organizations, or those of the publisher, the editors and the reviewers. Any product that may be evaluated in this article, or claim that may be made by its manufacturer, is not guaranteed or endorsed by the publisher.
