# Supplementary material for: Development of a Structured Query Language and Natural Language Processing Algorithm to Identify Lung Nodules in a Cancer Centre
Source: Front Med (Lausanne). 2021 Nov 4;8:748168. doi: 10.3389/fmed.2021.748168 (PMC8599820; doi:10.3389/fmed.2021.748168)
Supplement: Supplementary file 1 [file Data_Sheet_1.PDF]

SUPPLEMENTARY

Disease Group	Terms Within Coded Text
Breast	BREAST, MAMMARY, NIPPLE, IDC, DCIS, INFILTRATING DUCT, INVASIVE DUCTAL, INFILTRATING LOBULAR, LOBULAR CARCINOMA, LCIS, LOBULAR
CNS	MEDULLO, GLIOBLAST, EPENDYMOMA, PITUITARY, GLIOM, MENINGIOM, ASTROCYT, OLIGODENDRO
Colorectal	CRC, ANUS, SPLENIC FLEX, CECU, ANAL, COLO, ILEUM, JEJUN, BOWEL, DUODE, RECTAL, RECT, CAEC, CEAC, SIGMOID, APPEND
Germ Cell	GERM CELL, GERM, TERATOMA, SEMINOMA, TEST, YOLK
Gynaecological	OVAR, CERVI, VAGINA, PSEUDOMYXO, SEROUS, BARTHOL, VULVA, PERITONE, FALLOPIAN, GYNAE, ENDOMET, UTERUS, UTERINE
Haematological	bALL, B-CELL ALL, A.L.L, AML, CMML, MGUS, CML, CLL, FOLLIUCULAR, LEUKAEMIA, APML, MYELODYSP, DLBCL, DIFFUSE LARGE, MDS, B-ALL, T-ALL, ALL, PLL, LEUKEMIA, PLASMACYTOMA, LYMPHOMA, MALT, NHL, MYELO, HAEMATOLOGICAL, MARROW, BURKIT, HODGKIN
Head and neck	PAROTID, GLOTTIS, GLOTTIC, MOUTH, THYOI, LACRIMAL, VALLECU, GLOTTI, LARYNX, PIRIFORM, LARYN, SALIVARY, PHARYN, PALATE, BUCCAL, ORO, MANDIB, MAXIL, TONS, NASOP, NASAL, SINUS, BASE OF SKULL, SKULL BASE, VOCAL, RETROMOL, CRICOID, ORAL, HEAD, NECK, TONGUE
Lung	LUNG, PLEURA, MESOTHEL, HILAR, THYM, BRONCH, UPPER LOBE, LOWER LOBE, MIDDLE LOBE
Neuroendocrine	ENDOCRINE, CARCINOID
Sarcoma / Stromal Tumours	SARCOMA, PERIPHERAL NERVE, MPNST, DESMOID, GIST, SMOOTH MUSCLE, OSTEOSAR, STROMAL, EWING, SPINDLE, FIBROUS, RHABD, MYOFIB
Skin	MELANOMA, BASAL CELL, SKIN, DERMIS, BCC, B.C.C, BOWENS, SCALP, EAR, MERKEL,
Upper GI and HPB	STOMACH, GASTRIC, HCC, CHOL, PANCREA, ESOPH, PYLOR, BIL, AMPUL, HEPAT, OG JUNCTION, O-G, GOJ, OGJ
Urological	PROSTATE, RENAL CELL, RENCAL ANCER, KIDNEY, BLADDER, URETER, URO, TCC, TRANSITIONAL, EPIDID, Bladder, bladder, PENI, NEPHR, URACHUS, URETH, WILM

Supplementary Table 1: Patient disease groups were attributed by searching clinical-coder disease entries for the provided terms, using the R ‘grepl’ function.

	Suspicious After (%)	Suspicious Before (%)	Total
Breast	73 (69)	33 (31)	106
Colorectal	57 (69)	26 (31)	83
Germ Cell	56 (92)	5 (8)	61
Gynaecological	56 (53)	50 (47)	106
Haematological	3 (43)	4 (57)	7
Head and neck	15 (71)	6 (29)	21
Lung	29 (42)	40 (58)	69
Other	9 (69)	4 (31)	13
Sarcoma / Stromal Tumours	47 (68)	22 (32)	69
Skin	30 (77)	9 (23)	39
Upper GI and HPB	25 (64)	14 (36)	39
Urological	36 (67)	18 (33)	54

Supplementary Table 2: In patients developing metastatic disease after a lung nodule diagnosis, the proportion with a suspicious flag preceding or following the metastatic diagnosis are shown (n = 667).

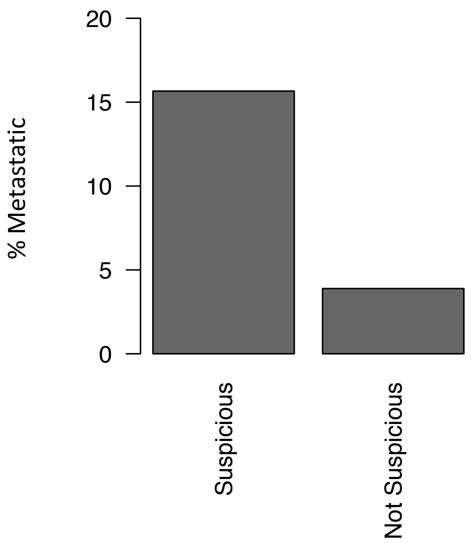

Supplementary Figure 1: Proportion of patients developing metastatic disease by suspicious status are shown (n = 1019 patients developing metastatic disease after a nodule diagnosis)
